# Pattern recognition receptors as potential therapeutic targets in inflammatory rheumatic disease

**DOI:** 10.1186/s13075-015-0645-y

**Published:** 2015-05-15

**Authors:** Lisa M Mullen, Giselle Chamberlain, Sandra Sacre

**Affiliations:** Brighton and Sussex Medical School, Falmer, Brighton, BN1 9RY UK

## Abstract

The pattern recognition receptors of the innate immune system are part of the first line of defence against pathogens. However, they also have the ability to respond to danger signals that are frequently elevated during tissue damage and at sites of inflammation. Inadvertent activation of pattern recognition receptors has been proposed to contribute to the pathogenesis of many conditions including inflammatory rheumatic diseases. Prolonged inflammation most often results in pain and damage to tissues. In particular, the Toll-like receptors and nucleotide-binding oligomerisation domain-like receptors that form inflammasomes have been postulated as key contributors to the inflammation observed in rheumatoid arthritis, osteoarthritis, gout and systemic lupus erythematosus. As such, there is increasing interest in targeting these receptors for therapeutic treatment in the clinic. Here the role of pattern recognition receptors in the pathogenesis of these diseases is discussed, with an update on the development of interventions to modulate the activity of these potential therapeutic targets.

## Innate immunity

The mammalian immune system consists of two effector arms: the innate non-specific arm and the adaptive arm, which recognises pathogens in an antigen-specific manner. These two components of the immune system have evolved to work in concert to provide a comprehensive defence against a wide variety of pathogens, including bacteria, viruses and fungi. The innate immune system provides an immediate response in an effort to limit the systemic spread of infectious agents. To do this the receptors of the innate immune system need to be able to identify a wide range of pathogens. This is made possible by recognition of evolutionarily conserved pathogen-associated molecular patterns (PAMPs) and as such these receptors are termed pattern recognition receptors (PRRs) [1].

To enable comprehensive surveillance for pathogens, PRRs are expressed as soluble receptors, on the cell surface, in the cytosol and in the endosomal compartments of cells (Table 1). The key functions of PRRs are to upregulate cell surface markers to trigger adaptive immunity and to induce the expression and release of cytokines, which activate tissue-resident macrophages and recruit further immune cells to the site of infection. A similar response occurs during many chronic inflammatory diseases and tissue damage, where in a sterile inflammatory environment PRRs are activated by their ability to respond to danger signals often referred to as ‘damage-associated molecular patterns’ (DAMPs) [2]. These are endogenous host molecules that are released from stressed or dying cells or that have formed crystals due to their presence in high concentrations - for example, monosodium urate (MSU) crystals in gout [3].

**Table 1:** **Pattern recognition receptors associated with rheumatic disease**

		**Pattern recognition receptor ligand(s)**		**Association with rheumatic**
**Receptor**	**Localization**	**PAMPs**	**DAMPs**	**disease**
**TLRs**				
TLR2	Cell surface	Lipoproteins	Gp96	RA [37], OA [50]
		Peptidoglycan	Hyaluronan	
		Zymosan	HMGB1	
			SNAPIN	
TLR3	Endosome	dsRNA	DNA	RA [24,30-32], OA [56]
TLR4	Cell surface	LPS	HSP70	RA [20,32,92], OA [42,43,48,50]
		Envelope glycoproteins	HMGB1	
		Mannan	Hyaluronan	
			Fibronectin	
			Biglycan	
TLR5	Cell surface	Flagellin		RA [34]
TLR7/8	Endosome	ssRNA	ssRNA	RA [25-27], SLE [93,94]
TLR9	Endosome	CpG DNA	DNA	OA [57], SLE [95]
**NLRs**				
NOD1	Cytoplasm	Diaminopimelic acid		RA [39]
NLRP3	Cytoplasm	RNA, DNA, MDP	ATP	Gout [3], RA [40], OA [59,60]
			HA/uric acid crystals	

Some examples of both exogenous (PAMPs) and endogenous (DAMPs) ligands are shown for each receptor. DAMP, danger-associated molecular pattern; dsRNA, double-stranded RNA; HA, hyaluronic acid; HMGB-1, high mobility group box-1; LPS, lipopolysaccharide; HSP70, heat shock protein-70; MDP, muramyl dipeptide; NLR, NOD-like receptor; NOD, nucleotide-binding oligomerization domain; OA, osteoarthritis; PAMP, pattern-associated molecular pattern; RA, rheumatoid arthritis; SLE, systemic lupus erythematosus; SNAPIN, SNAP-associated protein; ssRNA, single-stranded RNA; TLR, Toll-like receptor.

## Mammalian pattern recognition receptors

**Figure 1:**
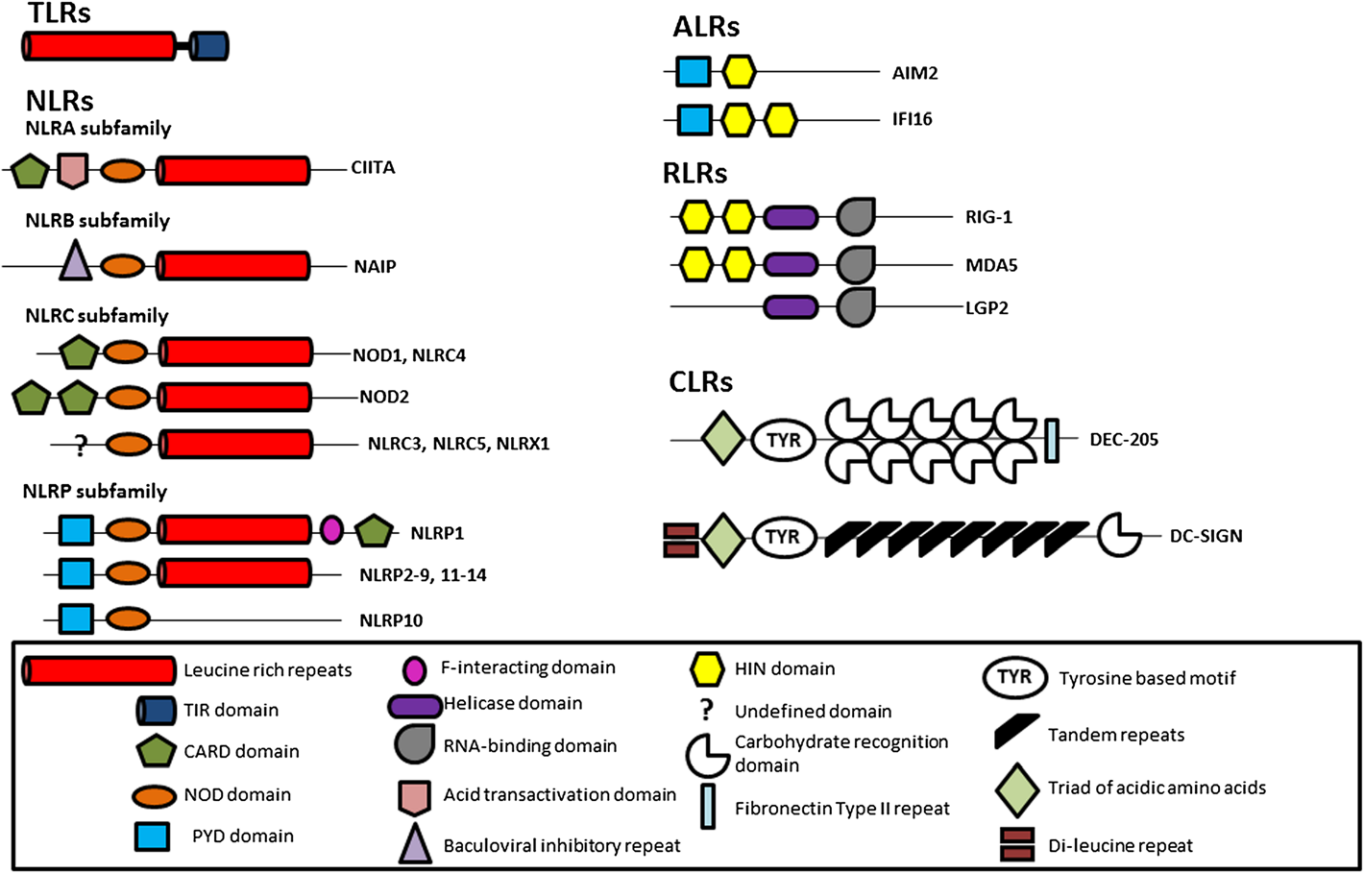
Schematic representation of the major mammalian pattern recognition receptor families. Common to all Toll-like receptors (TLRs) are leucine-rich repeats and a Toll-interleukin 1 receptor (TIR) domain. Nucleotide-binding oligomerization (NOD)-like receptors (NLRs) are characterised by a central NOD domain and the defining feature of RIG-I-like receptors (RLRs) is a helicase domain. The two members of the recently described AIM2-like receptors (ALRs) have HIN domains in common. The C-type lectin receptors (CLRs) are a very diverse group of proteins with no common domain structure. The type I (DEC-205) and type II (DC-SIGN) transmembrane CLRs are represented here.

The PRRs are able to recognise a diverse range of PAMPs and DAMPS and are divided into five classes according to their structural homology (Figure 1). The best characterised in terms of known ligands, signalling pathways and functional biology are the Toll-like receptors (TLRs) [4]. These are type I transmembrane receptors, expressed either in the plasma membrane (TLR1, 2, 4, 5, 6, 10) or in the endosomal membrane (TLR3, 7, 8, 9). TLRs can respond to a wide range of ligands that include proteins, lipopeptides and nucleic acids such as single-stranded RNA, double-stranded RNA or CpG DNA. Once activated, the TLRs engage adaptor molecules to initiate downstream signalling pathways culminating in the activation of either interferon regulatory factor (IRF) family members or nuclear factor (NF)-κB. The differential usage of the four adaptor proteins, myeloid differentiation primary response 88 (MyD88), MyD88-adapter-like (Mal), TIR-domain-containing adapter-inducing interferon-β (TRIF) and TRIF-related adaptor molecule (TRAM), determines which pathways are activated by individual TLRs [5].

Another family of primarily membrane bound PRRs are the C-type lectin receptors (CLRs), some of which exist as soluble receptors, such as the mannose-binding lectin that activates the complement cascade. They are a disparate family of receptors that bind to carbohydrate structures in a calcium-dependant manner and are possibly the least well-characterised of the PRRs, in part due to their sheer diversity. The membrane bound CLRs exist as type I (for example, DEC-205 and the macrophage mannose receptor) or type II (Figure 1) transmembrane receptors (for example, dectin 1, dectin 2 and DC-SIGN) [6]. These receptors have important roles in antigen uptake and in modulation of innate immunity.

The remaining families of PRRs are intracellular, expressed in the cytoplasm. The largest family are the nucleotide-binding oligomerization domain (NOD)-like receptors (NLRs), of which there are 22 in humans [7]. NLRs are further divided into subclasses (Figure 1), the largest of which are the NLRCs, which contain a caspase recruitment domain required to activate NF-kB, and the NLRPs, which have a pyrin domain, which is essential for the induction and regulation of the caspase-1-inflammasome. A number of the NLRs are known to form inflammasomes, but physiologically relevant roles have so far only been shown for those formed by NLRP1, NLRP3, NLRC4 and NAIP5, the most well characterised of which is NLRP3. Upon activation, NLRP3 associates with its adaptor protein apoptosis-associated speck-like protein containing a caspase recruitment domain (ASC), and caspase-1 to form a large oligomeric protein complex. This leads to the activation of caspase-1, which can then cleave pro-IL-1β and pro-IL-18 to their mature forms to be released from the cell [8]. Activation of the NLRP3 inflammasome also results in pyroptosis releasing the inflammasome complex as a speck from the cell into the extracellular environment; specks can then be taken up by neighbouring cells, further triggering activation of the inflammasome in these cells [9].

The remaining classes of PRRs are cytosolic nucleic acid sensors. The cytosolic retinoic acid-inducible gene-1 (RIG-1)-like receptors are cytoplasmic RNA sensors; the family is composed of RIG-1, melanoma differentiation associated gene 5 (MDA5) and laboratory of genetics and physiology 2 (LGP2). Cytoplasmic DNA is recognised by the relatively new family of PRRs, the absent-in-melanoma 2-like receptors (ALRs), consisting of four proteins in mammals: gamma-interferon-inducible protein (IFI16), absent in melanoma 2 (AIM2), myeloid cell nuclear differentiation antigen (MNDA) and interferon-inducible protein X (IFIX) [10]. They all share a HIN200 domain that confers DNA-binding activity, as well as a pyrin domain that can associate with other pyrin-containing proteins to enable assembly of an inflammasome. However, only IFI16 and AIM2 have so far been demonstrated to induce inflammasome formation. In addition to ALRs, other cytosolic DNA sensors have been identified in recent years - for example, cGAS, which can activate interferon (IFN) production through activation of stimulator of IFN genes (STING) [11].

## The potential role of pattern recognition receptors in inflammatory joint disease

A growing body of literature has suggested a role for innate immune PRRs as key contributors to several chronic inflammatory diseases where inflammation plays a major role in maintaining the chronicity of the disease and exacerbating the symptoms. In particular, TLRs and NLRs have the potential to drive the chronic inflammatory component of many arthritides, such as ankylosing spondylitis, psoriatic arthritis, systemic lupus erythematosus (SLE)-associated arthritis, rheumatoid arthritis (RA), osteoarthritis (OA) and gout in a sterile setting. However, as our knowledge of the other PRR families expands other families may also be found to be of importance. Consequently, these receptors or their signalling pathways are being explored as novel therapeutic targets for RA, OA, gout and SLE, which will be discussed in this review. A number of therapeutics, including small molecules based on targeting TLRs and NLRs, are already in development [12] (Table 2). The NLRs are particularly amenable to targeting with small molecule inhibitors due to the presence of the NOD domain, an ATP-binding domain that may facilitate competitive or allosteric inhibition.

**Table 2:** **Developmental status of pattern recognition receptor antagonists for potential use as therapeutics**

**Compound**	**Target**	**Drug class**	**Indications**	**Company**	**Clinical phase**
NI-0101	TLR4	Antibody	RA	Novimmune	Phase I
Chaperonin 10/X Toll	TLR4	Protein	RA, psoriasis, MS	Cbio Ltd	Phase II
VTX-763	TLR8	Small molecule	Autoimmune disorders	VentiRx Pharmaceuticals	Pre-clinical
CRID3	NLRP3	Small molecule		Amgen	Pre-clinical
OPN-305	TLR2	Antibody	Ischaemia reperfusion injury	Opsona Therapeutics	Phase I
IMO-3100	TLR7/TLR9	DNA-based small molecule	Psoriasis	Idera pharmaceuticals	Phase II
DV1179	TLR7/TLR9	Small molecule	SLE	Dynavax	Phase II
CPG52364	TLR7/TLR9	Small molecule	SLE	Pfizer	Phase II

MS, multiple sclerosis; NLR, nucleotide-binding oligomerization domain-like receptor; RA, rheumatoid arthritis; SLE, systemic lupus erythematosus; TLR, Toll-like receptor.

## Rheumatoid arthritis

RA is characterised by infiltration of inflammatory cells and fibroblast proliferation in the synovial joints, leading to chronic inflammation and a progressive destruction of bone and cartilage [13]. The cells in the RA joint produce elevated levels of cytokines, including TNF, IL-1 and IL-6, that support Th17 cell differentiation and suppress the differentiation of regulatory T lymphocytes, further perpetuating the inflammatory environment [14]. The past decade has seen the treatment of RA transformed by the use of biological therapies such as anti-TNF. Whilst a clear improvement over conventional treatments such as methotrexate, many patients fail to respond adequately to anti-TNF and can become unresponsive to treatment over time. More recently tociluzimab, an IL-6 receptor antibody, has shown great promise in this patient group [15]. However, the manufacturing cost of biological therapies remains a factor that severely restricts their use. On cessation of these therapies the disease reactivates in time, demonstrating that cytokine blockade does not modify the upstream disease mechanisms but instead works by dampening the resulting inflammation.

The pathogenic mechanisms are still poorly understood. There is increasing evidence from studies in both human and animal models of RA that TLRs and NLRs may contribute to this process [16,17]. Although it has long been proposed that infection could play a role in the initiation of RA, no specific pathogen has ever been linked to disease. However, the identification of elevated levels of endogenous PRR ligands, such as HMGB1, fibronectin and, more recently, the heat shock protein GP96, in the RA joint has highlighted the possibility that activation of PRRs may play an important role in the maintenance of inflammation in RA [18].

A variety of animal models have been used to identify the potential roles of TLRs in RA with mixed results. IL-1 receptor antagonist-deficient (IL-1Ra^−/−^) mice develop spontaneous arthritis, but when crossed with TLR4^−/−^ mice, disease severity is reduced [19]. These results are consistent with work from the same group demonstrating that a naturally occurring TLR4 antagonist from Bartonella Quintana had therapeutic effects in both the IL-1Ra^−/−^ model and the collagen-induced arthritis (CIA) model [20]. Conversely, TLR2^−/−^ mice crossed with IL-1Ra^−/−^ mice produced more severe arthritis, whilst crossing with a TLR9^−/−^ mouse had no effect on disease [19]. In the IL-1Ra^−/−^ mice TLR2 appears to be protective whilst TLR4 is linked with disease development. However, in a streptococcal cell wall-induced model of arthritis, TLR2-deficient mice show a reduced disease severity, highlighting the variability of results between arthritis models [21].

Conflicting roles have also been reported for TLR3. Activation of TLR3 suppressed arthritis in the mouse CIA and K/BxN serum transfer models [22]. However, TLR3 activation increased disease severity in the rat pristane-induced arthritis and rat CIA models, where upregulation of TLR3 was associated with disease and downregulation via small interfering RNA improved disease symptoms [23]. In a recent study, treatment of pristane-induced arthritis rats with a miR-26a mimic decreased TLR3 expression and disease symptoms, confirming a role for TLR3 in this model [24]. TLR7 has also been suggested to drive disease maintenance in the rat CIA model where intra-articular knockdown of TLR7 decreased disease activity [25]. In agreement with this study, we found that although TLR7^−/−^ CIA mice developed arthritis symptoms, the disease did not progress, leading to a reduction in clinical score and paw swelling compared with wild-type mice [26]. Together these studies suggest the potential for a contribution from TLRs to the maintenance of inflammation in arthritis models. However, there are many conflicting animal model studies in the literature, highlighting the need for caution when interpreting these results in relation to human disease.

Many human studies have been conducted on synovial tissue removed from patients during joint replacement surgery. Human RA synovial tissue is composed of a mixed population of cells, including RA synovial fibroblasts (RASFs), macrophages, T lymphocytes, B lymphocytes and dendritic cells, and has been demonstrated to be auto-stimulatory in culture. Expression of most of the TLRs has been demonstrated in the RA synovial membrane. In our own studies, we have demonstrated that activation of RA synovial membrane cultures with ligands for TLR1/2, 2/6, 3, and 4 and particularly TLR8 increased TNF production [27,28]. The major cell type in the synovium is the RASFs expressing mainly TLR2, 3, 4 and 9, which on stimulation of TLR3 or 4 can promote Th1 and Th17 cell expansion [29]. Interestingly, IL-17 has been reported to increase the expression of TLR2, 3 and 4 in RASFs, creating the potential for a positive feedback [30].

In comparison to OA tissue where there is a lower level of inflammation, TLR2, 3, 4, 5, 7 and 9 are known to be expressed at elevated levels in RA tissue [31-34]. TLR5 expression is also elevated on RA peripheral blood monocytes and correlates with disease activity [34]. In addition to elevated expression of TLRs in the RA synovium, we have previously demonstrated a functional role for TLRs in generating inflammation in the RA synovium from data generated using dominant negative versions of the TLR adaptor proteins MyD88 and Mal. These dominant negative adaptor proteins prevent downstream signalling and lead to a significant reduction in spontaneous cytokine and matrix metalloprotease production from RA synovial membrane cultures [28]. Furthermore, we were also able to inhibit spontaneous cytokine production from RA cultures by addition of chloroquine or mianserin, both small molecule inhibitors of the endosomal TLR3, 7, 8 and 9 [35]. Mianserin is thought to inhibit TLRs via an off-target mechanism and as such does not represent a suitable drug for clinical trial; however, other inhibitors of the endosomal TLRs are being developed for SLE, such as DV1179 (Dynavax, Berkeley, CA, USA) and CPG52364 (Pfizer, New York, NY, USA), which may also be of therapeutic benefit in RA (Table 2). Anti-malarials such as hydroxychloroquine have long been used to treat RA and it is believed that their effects are mediated through inhibition of the endosomal TLRs. However, in clinical practice they are often given in combination with other therapies as they cannot be used at high doses due to ocular toxicity. Indeed, other anti-rheumatic agents traditionally used in the treatment of RA have since been shown to possibly work via modulation of TLR function. For example, auranofin, an organogold compound, has also been shown to inhibit TLR4 dimerization and TLR3-dependent TRIF signalling [36]. Another approach to inhibiting TLR function in RA is by neutralising antibodies (biologicals); Opsona Therapeutics (Dublin, Ireland) have developed a TLR2 neutralising antibody which they have shown can reduce spontaneous cytokine production from RA synovial explants [37]. However, this antibody is currently in clinical trials for other conditions and not yet for RA (Table 2). There are now several immune modulators at various stages of development that target TLRs with a potential role in RA (Table 2); though as yet none have been approved for use in the clinic.

In addition to TLRs, evidence is emerging that the NLRs may also have a role and could also represent potential therapeutic targets for RA [38]. Both NOD1 and NOD2 are expressed in synovial tissue and expression of NOD1 is increased in RA synovium compared with OA [17]. Silencing of NOD1 in RASFs decreased TLR2- and IL-1β-induced IL-6 production, suggesting that NOD1 can synergize with TLRs in potentiating inflammation in RA [39]. More recently, the NLRP3 inflammasome has been implicated in a murine model of RA, where deletion of A20 triggers spontaneous erosive polyarthritis through an increase in NLRP3 activation [40]. Several inhibitors of the NLRP3 inflammasome are currently in preclinical development and will be discussed below in more detail in relation to gout.

## Osteoarthritis

OA is the most common form of arthritis and a leading cause of musculoskeletal pain and disability worldwide. The normal balance of the turnover of articular matrix is upset, with a loss of collagen and aggrecan, which is due in part to the activity of aggrecanases and the collagenase MMP-13 [41]. However, the complex pathogenesis of OA is poorly understood and consequently there is a lack of clinically effective treatments, which means that many patients eventually go on to have joint replacement surgery. Until recently, OA was considered to be a disease primarily affecting cartilage and bone. However, synovitis is increasingly considered to be an important process in the pathogenesis, albeit at a much lower level than observed in RA. In OA patients, synovial inflammation has been associated with higher pain scores and more rapid destruction of cartilage [41].

Similar to RA, many endogenous molecules are present in the OA joint where they are suggested to function as DAMPs that can activate PRRs. These molecules are possibly released due to trauma and age (both strong risk factors for OA) or as a result of synovial and cartilage catabolism [42-44]. Obesity is another strong risk factor for OA and is associated with increased circulating levels of free fatty acids. The saturated free fatty acid palmitate can activate TLR4, and in synergy with IL-1β can induce chondrocyte death, release of IL-6 and extracellular matrix degradation [45]. Higher levels of TLR DAMPs such as tenascin C, HMGB1, fibrinogen and fibronectin isoforms have been observed in OA synovial fluid and tissue samples [46-48]. Furthermore, fibrin, the cleaved form of fibrinogen, has been associated with disease severity in OA [49]. In addition to the presence of DAMPs, synovial fluid from patients with early OA has been shown to augment the responses of TLR2 and TLR4 in fibroblast-like synoviocytes through the provision of increased levels of the TLR accessory molecule CD14 [50]. CD14 is also suggested to act as an accessory molecule for TLR3, 7, 8 and 9, leaving the possibility that activation of other TLRs could also be amplified in OA joint tissue [51,52].

A variety of TLRs are expressed in cells within the joint. Articular chondrocytes express TLR1-9 at the mRNA level [53] and the proportion of cells expressing TLRs in the cartilage has been observed to increase with the grade of OA lesion [54]. TLR2, 3, 4 and 7 have also been detected in the synovium in OA [41,55]. Furthermore, functional polymorphisms in the promoters of TLR3 (rs3775296) and TLR9 (−1486 T/C) have been associated with susceptibility to knee OA in Chinese populations [56,57].

However, most studies have focused on TLR2 and 4 as most of the DAMPs identified are ligands for these receptors. TLR-deficient animal models of OA have given mixed results. In the collagenase-induced mouse model, TLR2 knockout increased the degree of OA, whereas in the destabilised medial meniscus model TLR2 deficiency did not affect the extent of disease [46]. In the medial meniscectomy model, dual knockout of both TLR2 and TLR4 offered only mild protection [46]. Furthermore, in the knee menisectomy model, TLR1, 2, 4 or 6 knockout or knockout of the TLR adaptor molecule MyD88 had no effect [58]. It is important to note that several of these models are not associated with significant synovitis or inflammation and thus may not accurately reflect the human form of disease.

In addition to TLRs, there may also be a role for crystal-induced activation of the inflammasome. The deposition of hydroxyapatite (HA) crystals occurs in around 60% of OA patients, and correlates with severity of cartilage degradation [59]. HA crystals have been shown to stimulate secretion of IL-1β and IL-18 from murine macrophages in a NLRP3 inflammasome-dependent manner [60]. IL-1β in turn promotes synovial inflammation and cartilage degeneration, with elevated IL-18 in the cartilage and synovial fluid known to correlate with OA disease severity [60]. ANK-deficient mice can be used as a model of HA crystal-induced arthritis; they have a spontaneous mutation in the *ank* gene resulting in deposition of HA crystals in synovial fluid and articular cartilage causing cartilage erosion and joint immobility [61]. NLRP3-deficient ANK^−/−^ mice exhibit decreased degeneration of cartilage as well as joint inflammation, suggesting a central role for the NLRP3 inflammasome in the pathogenesis of OA where HA crystals are evident [60].

Based on these observations, inhibition of IL-1β would seem to be an attractive therapeutic for OA, particularly as recombinant IL-1Ra (anakinra), a competitive inhibitor of the IL-1 receptor, and an anti-IL-1β monoclonal antibody (canakinumab) are already licensed for use in the clinic. Disappointingly, intra-articular injection of anakinra failed to produce any therapeutic benefit above that of placebo [62]. However, a small study involving just three patients with erosive OA has demonstrated clinical benefits after 3 months of daily subcutaneous anakinra injections [63]. It may be that IL-1 blockade has a place in some clinical subtypes of OA or may require combination with other therapies to be fully effective, but larger studies would need to be performed to determine this. Whilst there appears to be a potential role for TLRs and NLRP3 in the generation of the inflammation associated with OA, research in this area remains in its infancy. However, until there is a clear idea of which PRRs are of importance and the contribution that they make to the pathology of OA, it is unlikely that any of these inhibitors will be specifically investigated in the context of OA. Currently the treatment of OA lacks effective disease-modifying drugs, instead relying on nonsteroidal anti-inflammatory drugs to provide pain relief. PRRs may provide novel therapeutic targets in the future if a contribution to OA pathogenesis can be confirmed.

## Gout

Another form of arthritis associated with the production of IL-1β is gout. This is an inflammatory arthritis caused by precipitation of MSU crystals in the joint. It is distinguished from RA and OA by the clinical presentation of acute inflammatory monoarthritis, typically affecting the metatarsophalangeal joint. A defining feature of gout is acute inflammatory flares that develop very rapidly, reaching a peak 6 to 24 hours after onset, followed by spontaneous resolution within 4 to 14 days [64]. In the absence of effective long-term treatment, these recurrent acute attacks can progress to chronic disease resulting in an erosive polyarthritis [64]. Although a role for MSU in gout has been recognized for centuries, the mechanisms by which MSU crystals trigger the pain and inflammation of an acute gout attack have only more recently been unravelled. There is now compelling evidence implicating IL-1β as a key mediator of inflammation in gout, which in turn promotes the influx of neutrophils into the synovium and joint fluid [65]. The discovery of the inflammasome propelled forward the understanding of the pathogenesis of gout, with the importance of the NLRP3 inflammasome now well established. It has been demonstrated that MSU crystals are taken up by cells, triggering the assembly of the NLRP3 inflammasome and subsequent production of IL-1β [3]. This was confirmed using macrophages from mice deficient in the inflammasome components caspase 1, ASC or NLRP3 which failed to activate IL-1β in response to stimulation with MSU crystals [3]. More recently, the CLR Clec12a (also known as myeloid inhibitory C-type lectin-like receptor) has been identified as a receptor for MSU in human neutrophils. The role of this CLR in sterile inflammation appears to be one of negative regulation of the neutrophil-mediated inflammatory response [66,67]. Knockdown of Clec12a in a human neutrophil cell line resulted in enhanced IL-8 production and Ca^2+^ signalling and Clec12a knockout mice showed increased neutrophil influx in response to MSU crystals. However, it is interesting to note that Clec12a signalling does not affect the NLRP3 inflammasome pathway [67], so this CLR is an unlikely target for gout therapy.

Treatment for gout has remained unchanged for decades. Non-steroidal anti-inflammatory drugs remain the first line of treatment, followed by glucocorticoids. The main therapeutic strategy for gout remains focused on decreasing the production of uric acid by inhibition of the enzyme xanthine oxidase [68]. Two inhibitors of xanthine oxidase, febuxstat and allopurinol, are licensed for clinical use. In addition, oral colchicine, which suppresses MSU crystal-induced inflammasome assembly, has been used clinically for many years, long before the discovery of the NLRs. It is very effective in treating gout, but is poorly tolerated due to gastrointestinal side effects. The mechanisms of action of colchicine are still being elucidated, but it is known to have effects on Clec12a and NLRP3. Neutrophil adhesion and chemotaxis are inhibited via maintenance of Clec12a expression and inflammasome formation is blocked by prevention of ASC association with NLRP3 due to its effects on microtubule formation [66,69]. In recent years, with the advent of biological therapies such as anakinra, rilonacept (soluble IL-1 receptor) and canakinumab, research has focused on blocking IL-1β as a means of controlling the symptoms of gout. Early clinical studies of these IL-1 inhibitors demonstrated good efficacy and high tolerance [70], although an increased risk of infection caused by blockade of pro-inflammatory cytokines remains a consideration, together with the high cost of these biological therapies. A desirable alternative would be a small molecular weight inhibitor of NLRP3. A number of small-molecule compounds that inhibit activation of NLRP3 have been developed, including glyburide (albeit at high doses), parthenolide, Bay11-7082 and cytokine release inhibitory drug 3 (CRID3) [71,72]. However, with the exception of glyburide, which is used to treat type 2 diabetes, these compounds are still in preclinical testing.

## Systemic lupus erythematosus

SLE is a systemic autoimmune disease that involves most organs of the body, with a wide range of clinical manifestations, including arthritis, nephritis, skin rash, anaemia and lymphopenia [73]. It is characterised by B-cell autoimmunity, particularly to nucleic acids and their binding proteins, which in part arises due to defective clearance of apoptotic cells [74,75]. Phagocytosis of DNA and RNA immune complexes via Fcγ receptors leads to activation of PRRs and, in particular, TLR7 and 9 as they recognize RNA and DNA, respectively [76,77]. In fact, TLR7 and 9 are robust activators of B cells and dendritic cells stimulating autoantibody production and IFN-α, which are both characteristic of SLE [78,79]. IFN-α plays a critical role in the severity and progression of SLE [80]. A high percentage of SLE patients (in the order of 95% of children and 70% of adults [81]) have an ‘IFN signature’, which refers to the higher expression of many IFN-inducible genes in these patients.

Other cytoplasmic PRRs involved in the antiviral response have also been linked to SLE. Variants of the RIG-1-like receptor MDA5 have been associated with apoptosis, inflammation and autoantibody production and with increased susceptibility to SLE [82]. Thus, it is likely that multiple pathways of nucleic acid recognition are involved in the generation of IFN-α. Supporting evidence from the literature include a role for the PRRs AIM2 and DNA-dependent activator of IRFs (DAI). DAI was reported to be increased in both SLE patients and murine models of disease, whilst AIM2 correlated with disease severity [83,84]. In agreement with these data, AIM2-deficient mice demonstrate decreased susceptibility to lupus-like disease in murine models, via inhibition of macrophage activation and secretion of proinflammatory cytokines [83].

The importance of NLRs in the pathogenesis of SLE is less clear. Although some NLRs (for example, NLRP3) have been associated with immune responses to DNA, the downstream cytokines generated by activation of these receptors are IL-1β and IL-18. Although both of these cytokines are pro-inflammatory and can activate macrophages, neutrophils and T cells, they have not been directly implicated in SLE. At present, therefore, the most relevant of the PRRs for therapeutic targeting in SLE remain the TLRs because of the current pre-clinical and clinical evidence of their roles in SLE, as well as the existence of a number of small molecular weight antagonists currently in development.

Quinine-containing compounds have been used in the treatment of SLE for some time. These drugs prevent acidification and maturation of the endosome, thereby blocking TLR signalling as acidification is a prerequisite for endosomal TLR activation [85]. The efficacy of this treatment [86] thus provides further indication of the involvement of TLRs in the pathogenesis of SLE and justifies the on-going research efforts in developing new therapies based on targeting TLR7 and 9. As shown in Table 2, a number of therapeutics that target endosomal TLRs are at various stages of development for the treatment of SLE. This approach, based on blocking TLR7- and 9-induced IFNα, may make patients less prone to infections than global direct suppression of IFN-α using neutralising antibodies, and so may provide a better therapeutic strategy.

## Conclusion

There is a clear potential for targeting TLRs and NLRs for the treatment of inflammatory rheumatic diseases such as RA, OA, gout and SLE. Although these receptors have a key role in the defence against pathogens, redundancy between the families may permit therapeutic inhibition of some receptors without having to significantly compromise innate immune defences. It is promising to note that MyD88-deficient patients (that have lost signalling from all of the TLRs apart from TLR3), although susceptible to a number of pyogenic bacteria, showed normal resistance to most infections [87]. Thus, partial inhibition of a single receptor or group of receptors may not have a significant effect on immunity.

There is also the possibility that longer term inhibition of PRRs could have a negative impact on tissue remodelling and repair. TLRs in particular have roles in tissue repair and regeneration after tissue injury [88]. There are little available data on the roles of TLRs in tissue repair in the context of arthritic joints, but the importance of TLRs in tissue regeneration has been demonstrated in the lung [89], liver [90] and colon [91]. Once the importance of TLRs in joint tissue remodelling and repair becomes clear, it may be possible to adjust the timing and selectivity of therapeutics accordingly.

For gout the role of the NLRP3 inflammasome has been clearly defined and it will be of great interest to follow the development of NLRP3 inhibitors such as CRID3 that have the capability to be of significant clinical benefit during disease flares. The involvement of PRRs in the pathogenesis of OA is much less clear, with further research required to determine which PRRs are of importance. For RA there are encouraging data from both mouse models and human studies suggesting a potential role for TLRs, and in particular the endosomal TLRs in disease maintenance. Targeting the endosomal TLRs is particularly attractive as these receptors are amenable to modulation by small molecular weight compounds which could present a much cheaper alternative to the anti-cytokine biological therapies currently used in the treatment of RA.

## Abbreviations

ALR: absent-in-melanoma 2-like receptor
CIA: collagen-induced arthritis
CLR: C-type lectin receptor
CRID: cytokine release inhibitory drug
DAMP: damage-associated molecular pattern
HA: hydroxyapatite
IFN: interferon
IL: interleukin
IL-1Ra: IL-1 receptor antagonist
IRF: interferon regulatory factor
MSU: monosodium urate
NF: nuclear factor
NLR: NOD-like receptor
NOD: nucleotide-binding oligomerization domain
OA: osteoarthritis
PAMP: pathogen-associated molecular pattern
PRR: pattern recognition receptor
RA: rheumatoid arthritis
RASF: rheumatoid arthritis synovial fibroblast
SLE: systemic lupus erythematosus
TLR: Toll-like receptor
TNF: tumour necrosis factor
